# How to … Apply for a Research Fellowship in Clinical Education: The ‘Six Ps’ of Success

**DOI:** 10.1111/tct.70380

**Published:** 2026-03-04

**Authors:** Paul A. Tiffin, Katie Wardle, Bakita Kasadha, Gillian Vance, Asta Medisauskaite, Emma Kelley, Cecily Henry, Sophie Park, Peter Thompson, Katherine Woolf

**Affiliations:** ^1^ Hull York Medical School Hull UK; ^2^ University College London Medical School London UK; ^3^ Nuffield Department of Primary Care Health Sciences University of Oxford Oxford UK; ^4^ School of Medicine Newcastle University Newcastle Upon Tyne UK; ^5^ National Institute for Health and Care Research (NIHR) London UK

**Keywords:** academic careers, career development, research funding

## Abstract

In this article, we describe how educators can maximise their chances of success when applying for research fellowships in clinical education. We outline the ‘Six Ps’: Person, Place, Project, Public/Patient involvement (PPI), Preparation and Price; we also address common concerns and provide practical examples of ‘easy wins’.

## 1 Introduction

Clinical educators often face important questions about their practice, with little research evidence to guide them; however, research funders are increasingly willing to support applications from promising candidates developing research careers in important yet evidence‐scant areas such as clinical education. The National Institute for Health and Care Research (NIHR), a major English funder, supports an ‘Incubator for Clinical Education’, which fosters a growing multi‐professional clinical education research community, supporting those who otherwise may have lacked opportunities [1].

For research‐active clinical educators, a fellowship can be career‐changing, offering mentoring, protected time, supervision and bespoke training. However, fellowships are highly competitive and researchers in teaching‐focused departments may not consider applying. Despite investments, clinical education research infrastructure remains under‐developed and with relatively few role models, leading to concerns from clinical educators about competitiveness and lacking guidance to apply successfully. Researchers from nursing, midwifery and allied health professions, and relevant non‐clinical disciplines are especially under‐represented [2, 3].

In June 2024, we held an inclusive NIHR Academy‐funded event at University College London (UCL) to support applicants from under‐represented groups to apply successfully for fellowships [4]. Pre‐event, delegates reported uncertainty surrounding application processes, eligibility and competitiveness. Post‐event, they felt inspired by fellowship holders' personal experiences and practical advice, with at least one delegate subsequently securing funding.

Drawing on lessons from this event, we share practical, transferable guidance for clinical education researchers considering fellowship applications. We highlight how, by considering the ‘five Ps’ [5]: *Person*, *Place, Project*, *PPI, Preparation* and adding a sixth ‘P’, *Price*, fellowship candidates can maximise their chances of success.

**Box 1 | Which funders offer research fellowships**

Most major funders have fellowship schemes. Spend time looking at funder websites to ensure you and your idea are within the funder‘s remit—what type of research will they or will they not fund and who is eligible to apply?

For those on a clinical academic career path in the United Kingdom, CATCH (Clinical Academic Training and Careers Hub) has a map of funding available at different career stages from 11 national funders [6].

In England, the NIHR, funded by the Department of Health and Social Care, provides research fellowships for health and care researchers at all career stages, from pre‐doctoral to post‐professorial, for clinical and non‐clinical researchers alike. In other UK countries, fellowship schemes are offered by NHS Research Scotland, Health Care Research Wales and Health and Social Care Services in Northern Ireland (HSC).

Other government‐funded research fellowship programmes include the European Commission‘s Marie Skłodowska‐Curie Actions fellowships, the United States National Institutes for Health (NIH) ‘F’ series, the Canadian Institutes of Health Research (CIHR) Health Research Training Award Programs and UK Research and Innovation fellowships, all of which can be applied to clinical education topics.

Charitable organisations such as Wellcome and the British Academy, as well as medical research charities, also provide fellowships that can be used for research on clinical education topics.

Different funders have different remits and areas of strategic importance. It is therefore important that you understand the types of research that particular funders are interested in and target accordingly.

## 2 The Six Ps

### 2.1 Person

During our event at UCL, we heard concerns about the perceived need for extensive research experience. Fellowship applicants are often judged against peers in their profession and by career stage. However, some funders, such as the CIHR, NIH, Wellcome and the NIHR, increasingly assess an applicant's outputs (publications/grants) in relation to their likely available opportunities, given their background. As such, candidates from clinical education, where funding opportunities may be relatively scarce, need not always have obtained significant grant funding, even at postdoctoral level. Small grant funding for education projects and PPI activity from local organisations or national societies can demonstrate early research promise. For pre‐doctoral and doctoral fellowships, publications are not always essential. In general, an applicant's CV should show a trajectory that highlights how the fellowship will support their research career and leadership capacity. Demonstrating enthusiasm and commitment to a research career at this stage is important, and this can be evidenced through non‐peer–reviewed articles or presentations if peer‐reviewed publications are not yet available.

A common reason for unsuccessful personal fellowship applications is a weak personal development plan. Fellowship panels want to see how such plans benefit the ‘Person’. Usually, strong plans include formal and informal learning experiences, such as taught short courses relevant to your research programme and exchange visits with centres of excellence. The latter should show how the experience will facilitate networking and build valuable skills and knowledge. In general, training and development activities should focus on delivering your proposed research whilst supporting longer‐term career development. Most funders like to see activities beyond your own host institution, even at doctoral‐level.

### 2.2 Place

Funders usually look carefully at the academic environment in which the fellow will be based. Carefully selecting supervisors and mentors is essential. These do not all have to be senior researchers or clinical, but should collectively bring relevant expertise. Whilst mentors are usually not costed into fellowship proposals, academics are often keen to support promising researchers in their field. Do not just go for the ‘big names’; your supervisors and mentors need to have sufficient time to be able to commit to you and have experience of taking candidates through to successful PhD completion.

Being embedded in a centre (‘host institution’) that supports research and development is key, as are links with other groups providing expertise. Individuals or centres with track records of research funding and impactful publications may be especially promising.

A letter of support from your host institution should describe tailored opportunities for research and career development, such as mentoring, journal clubs and conference funding.

For more senior postdoctoral fellowships, many funders will be looking for longer‐term commitments from your host to your ongoing academic career. More concrete commitments result in stronger applications.

**Box 2 | Easy wins.**

Find research centres that have supported previous successful fellowships by searching funder websites for details of funded fellowships, or reach out to relevant research networks, for example, Incubator for Clinical Education, the Association for the Study of Medical Education or the International Association for Health Professions Education. Within centres, do contact current research fellows to ask them about their experience to date [7].

Your host institution should provide information about expertise and training opportunities they will provide you for your particular fellowship.

Reach out to centres or individuals from across disciplines to ask for support with specific resources or expertise, and request a letter of support from them (you can offer to provide a draft).

### 2.3 Project

Your research project must be achievable, appropriate to your career stage and be a vehicle for developing your research capabilities. If based in England, you can submit your idea to the Research Support Service (RSS) early for expert advice with NIHR or other fellowship applications [8].

Developing a research idea that is both ‘worth doing’ and ‘doable’ is probably the biggest challenge. The NIHR Incubator for Clinical Education website (clinicaleducationresearch.org) could provide inspiration, but the best ideas usually emerge from our practice and lived experience. Any inspiration must be situated in the existing literature and demonstrate a clear gap in evidence, and so read widely, especially relevant journals.

When considering the scope of your fellowship project, avoid trying to do everything. Feasibility studies, developing and validating new instruments, mixed‐methods or qualitative projects are plausible. A multi‐centre randomised trial may be too ambitious, but preparatory studies with a clear plan for seeking follow‐on funding may be suitable.

You should situate your research within your jurisdiction's current healthcare policy priorities. A credible pathway to impact needs to go beyond conference presentations and publications. For example, explain how you will engage and collaborate with policymakers during your fellowship. Articulate how patients and the public, including those from diverse and under‐represented groups, will benefit directly or indirectly (e.g., by improving the knowledge and skills of medical graduates). Funders increasingly expect evidence that issues of inclusion have been considered [9].

Finally, be clear what the specific deliverables of your project will be. Beyond peer‐reviewed publications, these could also include outputs such as a toolkit of training materials, new datasets for other researchers to use, training materials and/or policy recommendations.

**Box 3 | Easy wins.**

Avoid over‐complicated research plans. Discuss these with your supervisors/mentors so that you set out the case for your research—showing that this will address an important problem, and that the design is achievable for your level and Fellowship time frame, and you are clear what the project deliverables will be.

Follow relevant policy organisations to keep abreast of significant policy developments to ensure your project goals are aligned to national priorities.

Ask your proposed host centre where you will be based about what support they have for research impact, and ask your supervisors/mentors for advice.

If based in England, seek early help from the NIHR Research Support Service (RSS).

### 2.4 Patient and Public Involvement (PPI)

Clinical education research should have input from those who will ultimately benefit. Ensuring diversity of background and experience is essential. Contributors may include patients, carers, and relevant community or patient organisations. Whilst some studies aim to recruit condition‐specific patients, clinical education research can attract a wider range of interested public partners and should also involve other beneficiaries, such as learners, educators and clinical staff. When considering who to involve, the key is to be clear about the experiences and knowledge needed, involve those most likely to bring them [10] and be transparent about how their involvement has shaped the research [11].

PPI members should be appropriately remunerated. To reimburse PPI members at the application stage, there may be institutional funds available. For England‐based applicants, you can apply to the RSS for such resources.

**Box 4 | Easy wins.**

Involve diverse PPI contributors as early as possible to help identify priority areas for research, design the research and its outputs, and disseminate findings.

Consider the particular needs of those from different backgrounds who may need different types of support to contribute.

Be aware that payment and recognition practices may differ for specific groups of patient partners. For example, the Canadian Institutes of Health Research (CIHR) highlights that Indigenous Elders and Knowledge Keepers follow a distinct protocol. In addition to reimbursement of participation‐related expenses (e.g., travel, meals, accommodation), they are offered a flat rate for the day irrespective of the time spent on the activity, alongside a culturally meaningful gift (tea, blanket, scarf, etc.) [12].

Explain in your application how PPI input shaped your proposal and research inclusion will be considered. This is mandated by some funders.

Seek input from existing PPI panels or networks at your higher education institution or healthcare provider.

Some funders have small pots of funding to apply for to reimburse PPI contributors for their time and expertise in helping you prepare your application.

Within your application include details of the PPI activities throughout your research timeline and include adequate costings for PPI time and training.

### 2.5 Preparation

At our event, we heard concerns about the significant amount of time and effort needed to write a competitive application, especially when this is done alongside teaching and clinical duties. Writing a strong application takes considerable preparation but offers years of protected research time in return.

Learning to deal with rejection is also part of an academic career. Where circumstances allow, unsuccessful applications can be improved and re‐submitted, or adapted for alternative funding. The networking, thinking and writing involved in an application generate new contacts and ideas and help raise your profile. Setting a realistic timeline for the application process is essential. Invest in identifying the appropriate funder, the career stage at which to apply, eligibility criteria and submission deadlines. Check whether you can apply directly to the funder: some fellowships involve internal selection by universities.

Complete a profile on the funding portal/application system and double‐check the documents needed, including mandatory attachments/uploads which may not be obvious. Allow plenty of time for institutional sign‐off; these can take at least 1–2 weeks.

Set aside months to develop your research project and personal development plan. Circulate an initial one‐page outline to potential supervisors and mentors early to flag any fundamental flaws and continue to seek review. Avoid asking mentors to review or sign‐off on projects close to deadlines.

The quality of the writing is critical. Applications must be concise and accessible for non‐specialists. Readers should quickly grasp the problem, evidence gap and how you, as the applicant, will be equipped via the fellowship to address the issue (the ‘problem/gap/hook heuristic’ [13] can be helpful).

If you are shortlisted, arrange at least one mock interview, asking supervisors/mentors to help. University research offices, or, in England, the RSS may be able to coordinate such preparation. Expect to be asked about any aspect of your application: the research, PPI, impact and career plans, and to explain your proposal in lay terms.

**Box 5 | Easy wins.**

Learning to bounce back from rejection is a core academic skill. The application process is itself useful, and you may be able to reshape and resubmit an unsuccessful application.

Check funder websites carefully for eligibility, requirements and timelines. Early on, tell the host institution you are applying from when you are intending to apply and find out internal application timelines.

Register and open a profile within the funding application website as early as possible and double‐check that you have a checklist of everything you need to submit, including mandatory uploads or attachments, and separate CV information.

Allow several months for feedback rounds from supervisors and PPI contributors.

Search funder websites or ask university research coordinators to identify potential reviewers who have recent success with the funder and ask them if they will review your application and/or run a mock interview.

### 2.6 Price

Use institutional costing templates and funder guidelines to include all permissible costs. Justify all requested funding for research (e.g., software, travel, interview transcriptions, participant payment); salaries for applicants (full or part‐time—some will pay clinical salaries) and research support (e.g., a post‐doctoral researcher or administrator); dissemination (e.g., journal publication and conference fees); and PPI activities or development plan costs. You could ask successful fellowship holders if they are prepared to share their (anonymised) costings and justifications.

Under‐costed applications appear naïve or unfeasible, cannot usually be corrected later and risk being unfeasible.

## 3 Conclusion

Research fellowships may seem intimidating, but they are career‐changing and valuable for education, health services and the public. Clinical education researchers understand only too well the critical importance of our field to patient care and outcomes. The process of writing a fellowship application will help communicate this to funders, encouraging them to support our future research leaders in this vital area.

## Author Contributions

**Paul A. Tiffin:** conceptualization, writing – original draft, writing – review and editing. **Katie Wardle:** conceptualization, writing – original draft, writing – review and editing. **Bakita Kasadha:** writing – review and editing. **Gillian Vance:** writing – original draft, writing – review and editing. **Asta Medisauskaite:** writing – review and editing. **Emma Kelley:** conceptualization, writing – original draft, writing – review and editing. **Cecily Henry:** writing – review and editing. **Sophie Park:** writing – review and editing. **Peter Thompson:** writing – review and editing. **Katherine Woolf:** conceptualization, writing – original draft, writing – review and editing.

## Funding

This event was funded by the National Institute for Health and Care Research (AMEF‐2023‐001, NIHR Incubators‐02‐2022‐22) and hosted by an NIHR Academy Member.

## Disclosure

The information presented is that of the organiser(s) and co‐authors, and not necessarily those of the NIHR or the Department of Health and Social Care.

## Ethics Statement

The authors have nothing to report.

## Consent

The authors have nothing to report.

## Conflicts of Interest

Katherine Woolf reports research funding during the period from the NIHR (NIHR157268, NIHR209881, NIHR302856) and from The Sutton Trust. Gillian Vance was the founding lead for the NIHR Incubator for Clinical Education and sits on the Incubator executive group. She is also a panel member for the NIHR Academy Doctoral Research selection process. Peter Thompson is Director of Programmes and Impact at the NIHR Academy.

## Data Availability

The authors have nothing to report.
